# *Lactobacillus gasseri LG08* and *Leuconostoc mesenteroides LM58* exert preventive effect on the development of hyperuricemia by repairing antioxidant system and intestinal flora balance

**DOI:** 10.3389/fmicb.2023.1211831

**Published:** 2023-06-12

**Authors:** Lizhen Liang, Zihui Meng, Fei Zhang, Zhu Jianguo, Shuguang Fang, Qingang Hu, Xuna Tang, Yanan Li

**Affiliations:** ^1^School of Food Science and Pharmaceutical Engineering, Nanjing Normal University, Nanjing, China; ^2^Nanjing Stomatological Hospital, Affiliated Hospital of Medical School, Nanjing University, Nanjing, China; ^3^Department of Research and Development, Wecare-Bio Probiotics Co., Ltd., Suzhou, China

**Keywords:** hyperuricemia, probiotic, prevention, therapy, antioxidant system

## Abstract

**Introduction:**

Currently, hyperuricemia has shown a surprisingly rising trend, which attracts widespread attention due to potentially major health risks. Considering the inevitable side effects of long-term medicine, probiotics are emerging as potential therapeutics due to their ability to improve uric acid metabolism and superior safety.

**Methods:**

In our study, two strains of probiotics, *Lactobacillus gasseri LG*08 (LG08) and *Leuconostoc mesenteroides LM*58 (LM58) isolated from kimchi were evaluated for the prebiotic properties *in vitro* and uric-lowering effects *in vivo*. Here, hyperuricemia animal model and 16S rRNA gene amplicons analysis were further studied to investigate whether these probiotics exert different effects in prevention and treatment.

**Results:**

*In vivo* indicators and intestinal flora immunity revealed that both LG08 and LM58 significantly prevent the development and progression of hyperuricemia, repair the antioxidant system and maintain intestinal flora balance in healthy rats, especially LM58. After hyperuricemia was formed, although the effect of LG08 and LM58 could decrease the level of uric acid, the effect to reverse and repair antioxidant levels in the body was limited.

**Discussion:**

In our study, these findings have important implications for hyperuricemia prevention and therapy, and provided more mechanistic insights into the effect of probiotics in hyperuricemia.

## 1. Introduction

Chronic hyperuricemia is a huge threat to gout, kidney disease, inflammatory arthritis, cardiovascular disease, diabetes, dyslipidemia, and other metabolic syndromes (Neogi et al., 2015; Roumeliotis et al., 2019). Recently, due to the abnormal purine metabolism or the high intake of purine and protein-rich food, the incidence of hyperuricemia has shown an obvious rising trend, which attracted widespread attention (Cirillo et al., 2006; Lima et al., 2015). In China, the overall hyperuricemia prevalence in the Chinese adult population was 11.1% in 2015–2016, and it increased to 14% in 2019 (Zhang et al., 2021). Also, it has been reported that the prevalence of gout is 3% in the US (Chen-Xu et al., 2019). In clinic, there are two strategies to alleviate hyperuricemia, one is to promote the excretion of uric acid through kidney, the other is to inhibit the metabolism of purine to uric acid by the liver and small intestine, or the two strategies are combined to further decrease the level of the uric acid in serum (Sattui and Gaffo, 2016; Matsuo et al., 2020). Until now, three types of uric acid-lowering drugs including xanthine oxidase inhibitors, uricosuric drugs and uricase are commonly used in the treatment of hyperuricemia. However, the side effects of these drugs largely limited their long-term use in clinic, including allergic reactions, severe skin reactions, cardiovascular side effects, liver toxicity and kidney hypersensitivity reactions (Ishikawa et al., 2020). Therefore, developing efficient and side-effect-free strategies to alleviate hyperuricemia is extremely urgent, especially for long-term control of uric acid.

In healthy individuals, about 70% of UA is excreted by the kidneys, and the remaining 30% is decomposed by intestinal flora (Yanai et al., 2021). Since the intestinal flora is directly involved in food digestion and absorption, and studies have shown that the intestinal flora of gout patients is dysregulated, the role of intestinal flora in the pathogenesis of hyperuricemia has been emphasize (Guo et al., 2016). Therefore, the potential function of probiotics to relieve hyperuricemia has also gradually been investigated, such as *Lactobacillus, bifidobacterium*, etc. (Xiao et al., 2020; Cao et al., 2022). However, most studies focused on screening microorganisms with anti-hyperuricemia function, such as *Lactobacillus DM9218 and Lactobacillus paracasei S12*, which evaluated their ability to transform purines *in vitro* (Wang H. et al., 2019; Xiao et al., 2020). *Lactobacillus gasseri PA-3* were reported to efficiently decrease the level of uric acid in hyperuricemia rat models (Kano et al., 2018). In addition, other functional characteristics are also important for probiotics to exert the uric acid-lowering effect. For example, as a key enzyme in the production of uric acid, the activity of xanthine oxidase (XO) could be significantly inhibited by *Lactobacillus rhamnose-I21* and *Lactobacillus reuteri L20M3* to ameliorate hyperuricemia in rat models (Ni et al., 2021).

Besides, the ability to resist gastrointestinal digestion, colonize on the epithelial cells and resist against pathogenic bacteria can also contribute to the efficiency of lowering uric acid (van Zyl et al., 2020). Moreover, the antioxidant and anti-inflammatory effects of probiotics are also of great importance in their uric acid lowering effect (Wang et al., 2017; Sichetti et al., 2018). It is worth noting that current research is only limited to how probiotics reduce the uric acid level in hyperuricemia models, but whether probiotics can prevent the occurrence of hyperuricemia is none. Furthermore, the difference between their roles in prevention and treatment needs to be further identified.

The aim of this study was to investigate the two probiotics, *Lactobacillus gasseri LG08* (LG08) and *Leuconostoc mesenteroides LM58* (LM58) would play a positive role in preventive and therapeutic effects on hyperuricemia by influencing the intestinal state and oxidative stress. The two strains of probiotics isolated from kimchi were investigated, including their tolerance to gastrointestinal conditions, adhesion aibility, anti-pathogen ability and *in vitro* antioxidant capacity. Then normal rat models and hyperuricemia rat models were established to evaluate the preventive and urico-lowering effects of probiotics *in vivo*. Meanwhile, by evaluating the level of inflammation *in vivo*, it would clarify that if the antioxidant activity of probiotics was conducive to improving its urico-lowering effect. Based on these experiments, this study provides a promising strategy for the prevention and remission of hyperuricemia.

## 2. Materials and methods

### 2.1. Bacterial strains and culture

Pure strains of *Lactobacillus gasseri LG*08 (LG08) and *Leuconostoc mesenteroides LM*58 (LM58) were obtained from Wecare Probiotics Co., Ltd (China). The two probiotics were inoculated in De Man, Rogosa, Sharpe (MRS) medium, respectively, and then cultured in anaerobic incubator at 37°C for 24 h. Afterward, two probiotic samples were obtained by inoculating 10% (v/v) of activated bacterial solution to 250 mL MRS medium and incubated at 37°C for 18 h.

### 2.2. Probiotic characterization

#### 2.2.1. Acid and bile salts tolerance

1 mL of two probiotic samples were added to 9 mL of MRS broth containing 0.1% (w/v) pepsin and pH = 3, respectively, and incubated at 37°C for 1 h. After incubation, 100 μL of probiotic samples were spread plated on MRS agar medium for 24 h at 37°C. Then, the number of colonies was calculated to verify the acid resistance of probiotics. Similarly, 1 mL of two probiotic samples were added to 9 mL of MRS broth containing 0.1% (w/v) cow bile salt, respectively, and incubated at 37°C for 4 h. The evaluation of bilt salts tolerance was the same as that of acid tolerance.

#### 2.2.2. Bacterial surface hydrophobicity

The adhesion of bacteria to hydrocarbons was carried out according to the literature previously reported (Rodríguez-Sánchez et al., 2021). Briefly, the probiotic samples were centrifuged (1,000 × *g* for 3 min at 4°C) and re-suspended in 3 mL of Phosphate Buffered Saline (PBS, pH 7.2) at 10^8^ CFU/mL. The initial value of absorbance at 600 nm (A_0_) was determined. Then, 1 mL of xylene and chloroform were added to 3 mL of bacterial suspension and vortexed for 1 min. The mixed suspension was allowed to separate into two phases after standing for 20 min, and the absorbance of the water phase (A_1_) was measured at 600 nm. The results were calculated using the following equation:


(1) $$\mathrm{Hydrophobicity}\left(\%\right)=\left(1-A_{1}/A_{0}\right)\times 100$$

#### 2.2.3. Autoaggregation and coaggregation assays

Autoaggregation and coaggregation ability of probiotics were measured using a previously known method (Sadeghi et al., 2022), with slight modifications. Bacteria in logarithmic stage were collected by centrifugation (5,000 × *g* for 15 min at 4°C), washed twice with PBS solution (pH 7.2) and resuspended in the same solution. For the autoaggregation assays, 4 mL of the mixture was vortexed for 10 s and the initial absorbance (A_0_) was measured. Then, it was incubated at 37°C for 5 h and the absorbance (A_*5h*_) was measured. The assay was performed in triplicate. The result of autoaggregation was determined following the equation:


(2) Autoaggregation(%)=(1-A/5A)0×100

For the coaggregation assays, bacterial suspensions were prepared as described above. Equal volumes (2 mL) of probiotics and pathogenic bacteria (*Staphylococcus aureus*) were mixed and vortexed for 10 s. After that, the mixture was incubated at 37°C for 5 h and then the absorbance (A*path+prob*) was measured. The absorbance of pure probiotics (A*prob*) and pure pathogenic bacteria (A*path*) were also measured as control. The assay was performed in triplicate. The result of coaggregation was determined following the equation:


(3) $$\text{Coaggregation}\left(\%\right)=\frac{\left({\text{A}}_{\text{path}}+{\text{A}}_{\text{prob}}\right)/2-{\text{A}}_{\text{path}+\text{prob}}}{{\text{A}}_{\text{path}}+{\text{A}}_{\text{prob}}/2}\times 100$$

#### 2.2.4. Antioxidant activity

The antioxidant activities of probiotics samples and their metabolites were evaluated by the β-carotene-linoleate model system. The β-carotene-linoleate model system was referred to reference with slight modification. Briefly, 2 mg of β-carotene, linoleic acid (44 μL), and Tween-80 (0.2 mL) were mixed in 10 mL of chloroform. 5 mL of the mixture was taken for rotary evaporation at 40°C. Then, 100 mL of distilled water was immediately added to the evaporated mixture and stirred well to form an emulsion. 0.5 mL of probiotics samples or their metabolites were added to 4.5 mL of the emulsion and placed at 50°C in a water bath. As a control, 0.5 mL of water was added to the same emulsion. Next, an emulsion without β-carotene was further prepared according to the above method, and 0.5 mL water was added to this emulsion as blank. The initial absorbances of all samples were measured at 470 nm (*t* = 0 min). Then, the absorbances at 470 nm were measured every 30 min until the color of β-carotene disappeared in the control group. All data shall be measured in triplicate. The antioxidant activity (AA) of probiotics and metabolites was evaluated by bleaching of β-carotene using the following formula:


(4) $$\mathrm{AA}=\left[1-\left(A_{0}-A_{420}\right)/\left(A_{0}^{\prime }-A_{420}^{\prime }\right)\right]\times 100$$

Where *A*_0_ and *A*_0_’ are the absorbance values of the sample and control group at *t* = 0 min, respectively. *A*_420_ and *A*_420_’ are the absorbance values of the sample group and control group after incubation for 420 min.

### 2.3. Experimental design based on the animal model

All experimental animals in our study were approved by the Animal Research Ethics Committee of Nanjing University of Chinese Medicine, after that the relevant ethical regulations were followed (Number: 202106A054). SPF-grade Sprague Dawley (SD) male rats which were 8-week-old and weighed 240–260 g, were bought from Shanghai SLAC Laboratory Animal Co. Ltd. All rats were maintained at 24 ± 1°C and 60–70% humidity with a 12 h light-dark cycle. Before the experiment, the rats were habituated for a week and free access to chow and water.

In the experiment of probiotics preventing hyperuricemia, SD rats were randomly assigned into four groups (*n* = 12): (1) blank; (2) high uric acid model group (P-Model); (3) LG08 prevention (P-LG08); (4) LM58 prevention (P-LM58). The blank group and high uric acid model group fed with standard diet and normal saline, while the LG08 and LM58 prevention groups were given the standard diet and, respectively, gavaged with LG08 strain and LM58 strain for 21 days. On the eighth day, all groups (excluding blank) were given oteracil potassium and adenine for 14 days to induce hyperuricemia.

In the experiment of probiotics treating hyperuricemia, SD rats were also randomly assigned into four groups (*n* = 12): (1) blank; (2) high uric acid model group (T-Model); (3) LG08 treatment (T-LG08); (4) LM58 treatment (T-LM58). Unlike the prevention groups, on the day 0, all groups (excluding blank) were gavaged with oteracil potassium and adenine for 21 days to induce and maintain a high uric acid state. Once the hyperuricemia was formed, T-LG08 and T-LM58 groups were, respectively, gavaged with LG08 strain and LM58 strain for 2 weeks from the eighth day, whereas the blank and high uric acid model group were gavaged with the same volume of normal saline. The administration concentration of all groups was 0.5 mL of 1 × 10^9^ CFU probiotics. During the treatment, the body weight of rats was recorded every 2 days.

After the last administration, the rats were fasted for 12 h and anesthetized using diethyl ether. Blood samples were collected from the abdominal aorta and centrifuged at 1,500 × *g* for 10 min to obtain serum. Then, serum was used to determine the level of blood urea nitrogen (BUN), creatinine (CRE), uric acid (UA), malonaldehyde (MDA), total antioxidant capacity (T-ROC) and glutathione peroxidase (GSH-PX). Also, small intestine tissues were collected, and the concentration of IL-7, IL-6, and TNF-α were detected by Elisa assay. Moreover, Small intestine and colon tissues were used for western blot analysis, immunohistochemistry and hematoxylin and eosin (HE) staining. In the end, feces were collected and used for the analysis of gut bacterial community. Except for those used for HE staining, all samples were stored at −80°C until analysis.

### 2.4. DNA extraction, amplification, and 16S rRNA gene amplicons analysis

In order to determine the effect of probiotic prevention and treatment on gut microbiota in rats, the rats’ feces were chosen for 16S rRNA gene amplicons analysis. Microbial DNA was extracted using E.Z.N.A.^®^ Soil DNA Kit (Omega Bio-Tek, USA) according to the manufacturer’s instructions. The final DNA concentration and purification were determined by NanoDrop 2000 UV-vis spectrophotometer (Thermo Scientific, 929 N Front St, Wilmington, NC, USA), and DNA quality was checked by agarose gel electrophoresis. The forward primer 338F: ACTCCTACGGGAGGCAGCAG and reverse primer 806R: GGACTACHVGGGTWTCTAAT were used to amplify the V3-V4 hypervariable regions of the bacteria 16S rRNA gene (Wang W. et al., 2019). The Illumina MiSeq platform (Illumina, San Diego, CA, USA) was performed to sequence paired-end purified amplicons according to the standard protocols. Statistical analysis was done following the packages from Qiime2docs along with customized program scripts.^1^ The sequences with identify greater than 97% were clustered into the same Operational Taxonomic Units (OTUs). Then, the OTUs were annotated by the Greengene database and used to calculate the number and percentage of bacteria in the samples (Bokulich et al., 2018). Alpha diversity index, such as Chao1 and observed OTUs, were calculated to investigate the microbial diversity in a single sample. Beta diversity was conducted based on the Bray Curtis, unweighted UniFrac and weighted UniFrac, which could be used to evaluate the structural variation of microbial communities across samples (Vázquez-Baeza et al., 2013). Then, the results were visualized by principal coordinate analysis (PCoA). Venn diagram was depicted to reveal the specific and common OTUs between different samples. Finally, Linear discriminant analysis effect size (LEfSe) was performed to detect differentially abundant bacterium.

### 2.5. Statistical analyses

Statistical analyses were performed using SPSS 22.0 (IBM SPSS, New York, USA) and GraphPad Prism 8. The data was presented as the mean ± s.d. The statistical significance was calculated via one-way ANOVA with Tukey’s multiple comparisons test.

## 3. Results

### 3.1. The physical properties of probiotics

**FIGURE 1:**
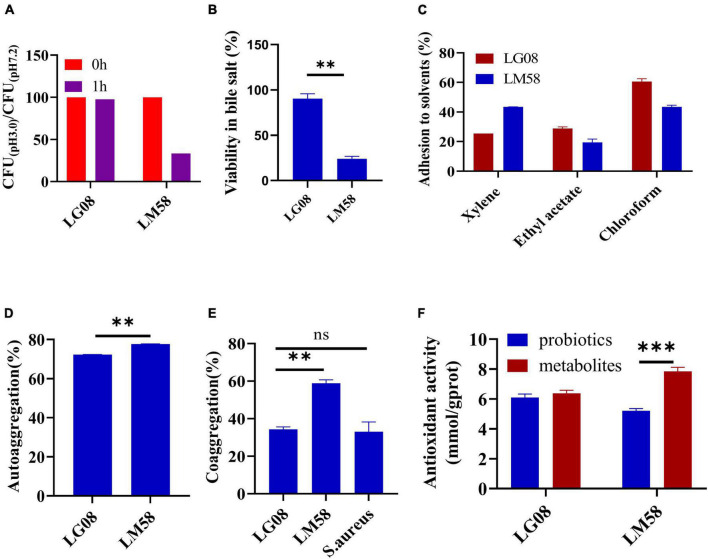
LG08 and LM58 exhibited different acid and bile salts tolerance, hydrophobicity, autoaggregation and coaggregation abilities and antioxidant activity *in vitro*. **(A)** The CFU ration of LG08 and LM58 in gastric juice (pH = 3.0) and normal pH 7.2; **(B)** The viability of LG08 and LM58 in bile salt (0.1% w/v) for 24 h; **(C)** The adhesion of LG08 and LM58 to xylene, ethyl acetate, and chloroform; **(D)** Autoaggregation ability of LG08 and LM58; **(E)** Coaggregation ability of LG08 and LM58; **(F)** Antioxidant activity of LG08 and LM58. The data are presented as the mean ± s.d., *n* = 3. The statistical significance was calculated via one-way ANOVA with Tukey’s multiple comparisons test. **p < 0.01, ***p < 0.001, ns means no significance. *S. aureus*, *Staphylococcus aureus*.

To explore the adaptability of probiotics in the gut, a series of evaluations for physical properties were conducted. Firstly, the tolerance to gastric juice and bile acid was important to maintain its activity in gut. As shown in Figure 1A, LG08 and LM58 showed 2% and 56% reduction in gastric juice after 1 h incubation, respectively. Next, LM08 and LM58 are able to survive after further incubation in bile acid solution for 3 h, while 10% reduction was observed for LG08 and 80% reduction for LM58 (Figure 1B). In Figure 1C, LG08 and LM58 both displayed low hydrophobic character, with less than 50% adherence to xylene. Moreover, the affinities with chloroform and ethyl acetate showed that LG08 and LM58 exhibited lower solubility (<25%) in ethyl acetate as well as a low affinity for chloroform (<90%), which were both characterized as Lewis acid. Referring to reports, pathogens also displayed a stronger affinity for chloroform (>95%), suggesting that these two strains were less hydrophobic than pathogens. Then, the aggregation ability of the probiotics under intestinal condition was investigated. LM58 showed higher autoaggregation and coaggregation ability than LG08 (Figures 1D, E), suggesting that LM58 could help to strongly antagonize the adhesion of pathogenic bacteria.

As high uric acid is closely related to oxidative stress in the body, the antioxidant effect of the two strains was determined by β-carotene bleaching methods. LG08 and LM58 showed 72 and 77% antioxidant activity (Figure 1F), which is consistent with previous studies. It is noted that the supernatant had a higher antioxidant activity than bacteria cells, especially for the LM58, indicating the secreted metabolites by probiotics have a strong antioxidant effect.

### 3.2. LG08 and LM58 prevent hyperuricemia development in healthy rat

**FIGURE 2:**
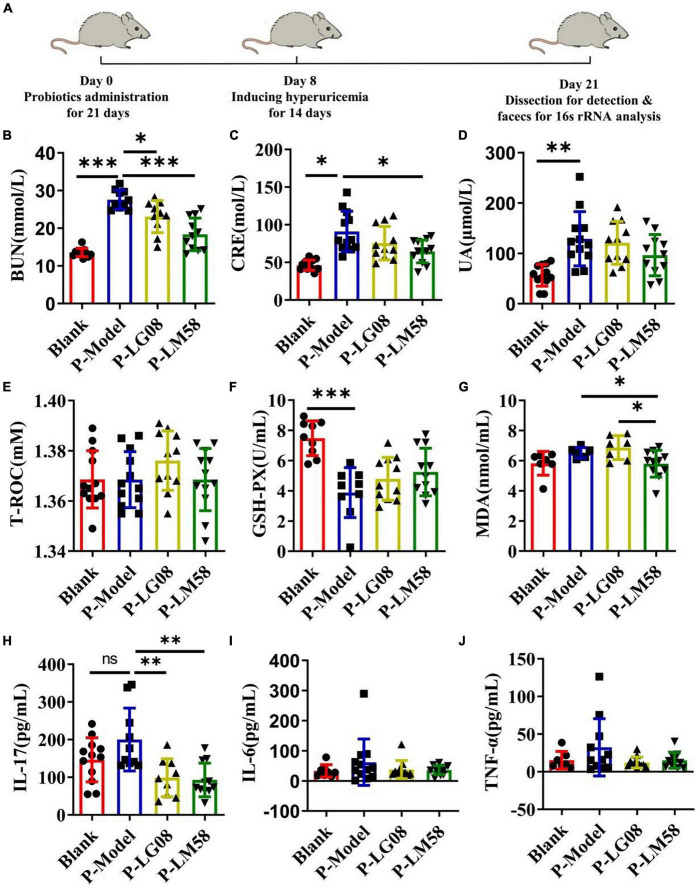
Feeding with LG08 and LM58 could reduce the levels of UA and other relative biochemical indexes in preventive hyperuricemia model mice. **(A)** The preventive process of LG08 and LM58 toward rat hyperuricemia model; **(B–D)** The levels of serum BUN, CRE, and UA in different rat groups (*n* = 6); **(E–G)** The levels of serum T-ROC, GSH, and MDA in different rat groups (*n* = 6); **(H–J)** The levels of IL-17, IL-6, TNF-α in small intestine (*n* = 6). The data are presented as the mean ± s.d., *n* = 6. The statistical significance was calculated via one-way ANOVA with Tukey’s multiple comparisons test. *p < 0.5, **p < 0.01, ***p < 0.001, ns means no significance. BUN, blood urea nitrogen; CRE, creatinine; UA, uric acid; T-ROC, total antioxidant capacity; GSH-PX, glutathione peroxidase; MDA, malonaldehyde.

To explore if the two probiotics could prevent the development of hyperuricemia in healthy rat administrated with high purine diet and the uricase inhibitor oteracil potassium, the level of UA and other relative biochemical indexes are tested after simultaneous oral administration of probiotics. The rat hyperuricemia model was constructed and the administration process was presented in Figure 2A. As shown in Figures 2B–D, the level of blood urea nitrogen (BUN), creatinine (CRE) and UA were all elevated after treating with high purine diet and the uricase inhibitor oteracil potassium (P-Model group), compared to blank group. When simultaneously treated with probiotics orally, the UA level in serum was 94% of that in P-Model group for P-LG08 group and 75% of that in P-Model group for P-LM58. As we can see, the level of BUN, CRE, and UA were all lower than these in P-Model group, suggesting that oral probiotics can prevent the development of hyperuricosis to some extent. Among that, the effect of LM58 is significantly stronger than that of LG08. Interestingly, there was no significant difference between UA levels of blank group and P-LM58 group, which demonstrated that LM58 could effectively prevent the formation of hyperuricemia.

Some reports found the activity of antioxidant enzymes in hyperuricemia rats induced by potassium oxazinate is significantly reduced, which resulted in the oxidative stress injury in the kidney and the rise of UA in blood. Besides, the abnormal level of UA could aggravate oxidative stress and further induce inflammation, which participate in the shape of inflammatory diseases such as atherosclerosis. Hence, evaluating the status of antioxidant ability is also important to reflect the potential to repair the oxidative damage in the body. As the main antioxidase system, T-ROC and GSH-PX in serum were examined. As shown in Figure 2E, there is no significant difference for T-ROC between these groups. However, GSH-PX levels of P-Model group decreased by about 50% compared with the blank group, suggesting the impairment of antioxidant capacity (Figure 2F). After intervention with LG08 and LM58, GSH-PX level showed slight recovery, reaching 63 and 70% of blank group, respectively, indicating that probiotics are likely to prevent the development and progression of hyperuricemia by repairing the antioxidant system, especially LM58. Finally, long-term hyperuricemia would accumulate gouty in the renal tubules, triggering a certain inflammatory response in the blood. The level of malonaldehyde (MDA), IL-17, IL-6, and TNF-α showed an increasing trend in the P-Model group (Figures 2G–J). Also, when compared with P-Model group, all these inflammatory markers were significantly induced. Specifically, these levels were kept similar to the control level after LM58 intervention. Based on the above results, LM58 plays a positively preventive role in the process of inhibiting the formation and development of hyperuricosis.

### 3.3. LG08 and LM58 prevent hyperuricemia development in healthy rat by regulating gut microbiota

**FIGURE 3:**
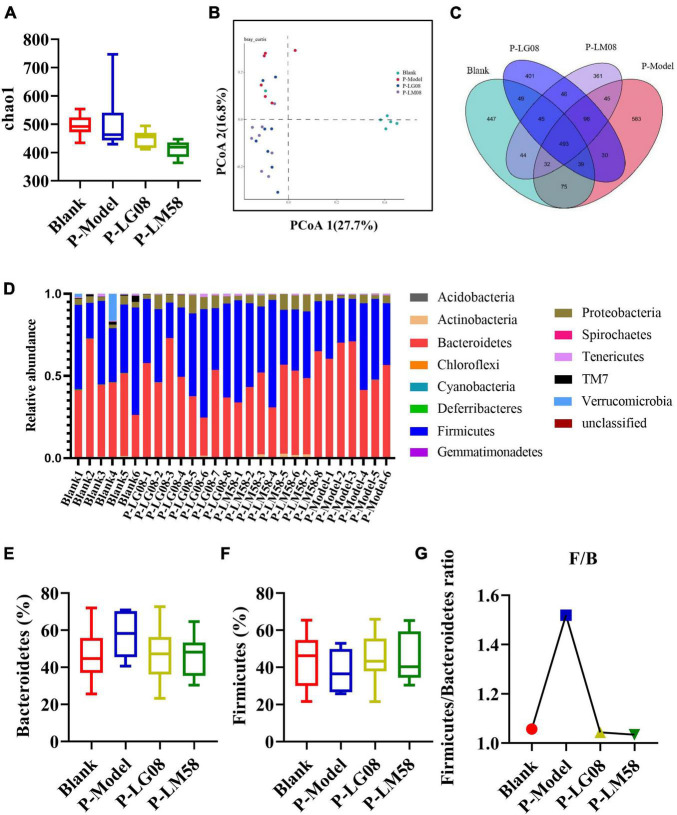
LG08 and LM58 prevented the development of hyperuricemia by maintaining the stability of intestinal microbial structure. **(A)** Chao 1 index based on the numbers of OTUs in the four samples; **(B)** The Principle coordinates analysis (PCoA) score scatter diagram of the four groups. PCoA of the overall diversity based on the Bray Curtis, unweighted UniFrac and weighted UniFrac; **(C)** The venn diagrams of the different groups according to bacterial biodiversity; **(D)** Comparison of phylum relative abundance in different groups; **(E,F)** Relative abundances of significantly changed bacterial phyla (Bacteroidetes and Firmicutes); **(G)** The Firmicutes/Bacteroidetes ratio in the different groups.

Recently, emerging studies have found that the gut microbiota of hyperuricemia patients had changed significantly compared with healthy people (Wei et al., 2022). To understand the relationship between gut microbiota and hyperuricemia, we performed the Miseq sequencing analysis of 16S rRNA to determine the diversity and abundance of gut microbiota in different groups. After treating with high purine diet and the uricase inhibitor oteracil potassium for 21 days, the abundance of microbial flora (chao 1) was slightly higher in P-Model group than in blank group (Figure 3A), which may be due to the administration of the high purine diet. After treatment with probiotics, the chao1 was lower than the P-Model group, especially for LM58. Principle-coordinates analysis (PCoA) results showed that phylogenetic community structures of P-Model group were markedly different from the blank group, and LG08 and LM58 groups also had a slight separation from the P-Model group, suggesting high purine diet and the uricase inhibitor oteracil potassium changed the gut microbiota composition, while the LG08 and LM58 could relieve the effect of gut changes to some extent (Figure 3B). Venn analysis further showed that P-Model group possessed a significantly different structure of the intestinal flora than blank group, and LG08 and LM58 could diminish the effect of P-Model group on microbiota changes (Figure 3C).

**FIGURE 4:**
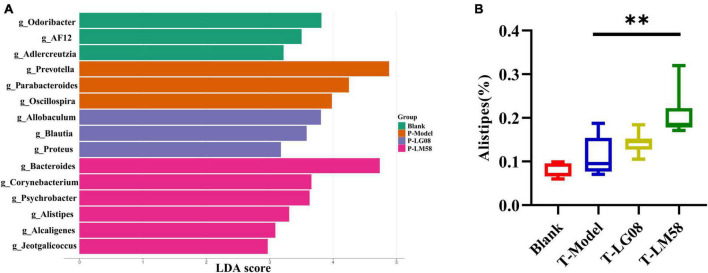
LG08 and LM58 prevention can affect the gut bacterial enrichment. **(A)** LEfSe cladogram of the four groups. **(B)** The relative abundance of *Alistipes* in the four groups. **p < 0.01.

Indeed, in patients with gout, the abundance of some bacteria decreased obviously, while others increased. For example, *Bacteroides caccae* and *Bacteroides xylanisolvens* were found enriched and *Faecalibacterium prausnitzii* and *Bifidobacterium pseudocatenulatum* decreased (Guo et al., 2016). Additionally, the ratio of *Firmicutes* to *Bacteroidetes* was considered to play an important role in maintaining a stable gut microbiota (Stojanov et al., 2020). From the relative abundance at the phylum level of each individual sample, the dominant phyla were *Bacteroidetes* and *Firmicutes* (Figure 3D). Comparing the P-Model group to blank group, there was a significant rise in the relative abundance of *Bacteroidetes* and a reduction in *Firmicutes*, while probiotic treatment kept that stable without significant difference (Figures 3E, F). Moreover, the ratio of *Firmicutes* to *Bacteroidetes* was also decreased from 1.5 to 1.0 with probiotic treatment (Figure 3G). Besides, the abundance of *Faecalibacterium*, *Bifidobacterium*, and *Roseburia* significantly has been proven to be increased in asymptomatic hyperuricemia (Yang et al., 2021). From the results of LEfSe cladogram in each group, the abundance of *Alistipes* in the P-LM58 group was significantly increased compared to the P-Model group (Figures 4A, B). It has been reported that *Alistipes* is an important stains in the gut that can produce short-chain fatty acids (SCFA) and reduce intestinal inflammation (Parker et al., 2020). This is consistent with the current research that intestinal flora can alleviate hyperuricemia by reducing inflammation and secreting small molecule metabolites, including short chain fatty acids. Hence, the LG08 and LM58 may prevent the development of hyperuricemia by influencing the intestinal microbial structure.

### 3.4. Therapeutic effect of LG08 and LM58 in hyperuricemia rat

**FIGURE 5:**
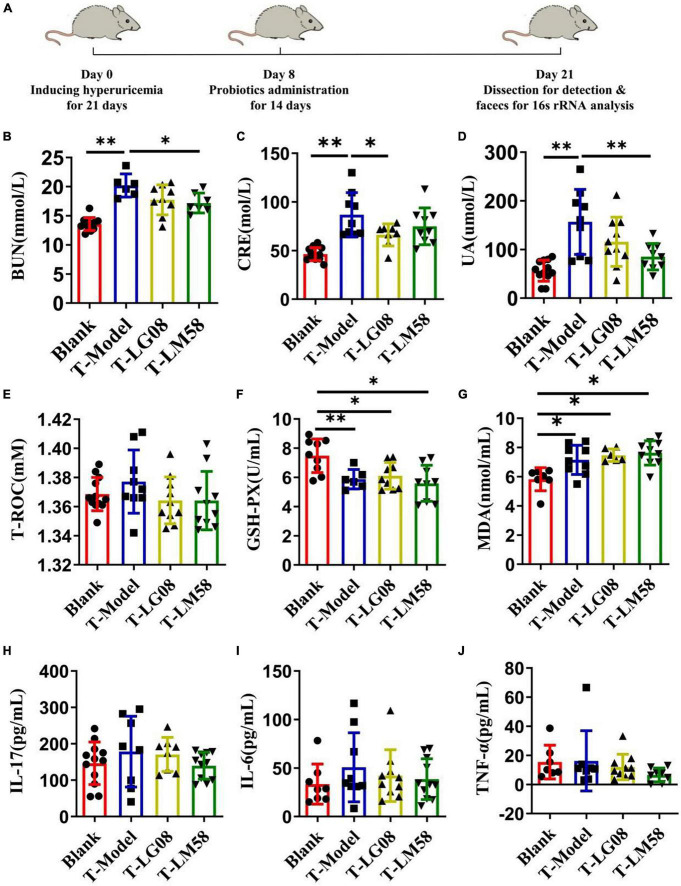
Feeding with LG08 and LM58 could reduce the levels of UA and other relative biochemical indexes in therapeutic hyperuricemia model mice. **(A)** The therapeutic process of LG08 and LM58 toward rat hyperuricemia model; **(B–D)** The levels of serum BUN, CRE, and UA in different rat groups (*n* = 6); **(E–G)** The levels of serum T-ROC, GSH-PX, and MDA in different rat groups (*n* = 6); **(H–J)** The levels of IL-17, IL-6, TNF-α in small intestine (*n* = 6). The data are presented as the mean ± s.d., *n* = 6. The statistical significance was calculated via one-way ANOVA with Tukey’s multiple comparisons test. *p < 0.5, **p < 0.01.

The previous study proved that LG08 and LM58 could prevent the formation and development of hyperuricemia in healthy rats, especially for LM58. To further test its therapeutic ability in hyperuricemia rat, the hyperuricemia model was firstly constructed and then the probiotics were treated orally (Figure 5A). As shown in Figures 5B–D, the LM58 could decrease the BUN level slightly and LG08 could decrease the CRE level. Similarly, the LG08 and LM58 both decreased the UA level, approaching 75 and 50% of that for T-Model group, respectively, indicating these probiotics could also reverse the symptoms of hyperuricemia. However, the two strains both cause no influence on the T-ROC and GSH-PX in hyperuricemia rat (Figures 5E, F). Besides, in hyperuricemia rat, the inflammatory factors were significantly increased, but there was no significant difference among blank group, T-LG08 and T-LM58 groups, suggesting that the two strains failed to restore the inflammatory level to the control (Figures 5G–J).

**FIGURE 6:**
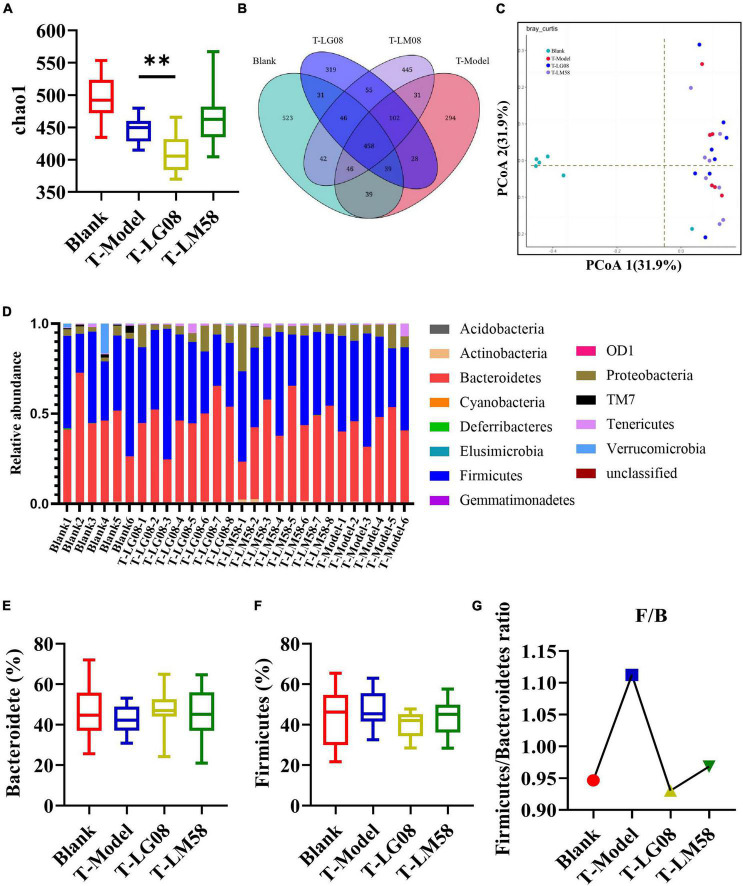
LG08 and LM58 treated hyperuricemia by maintaining the stability of the intestinal microbial structure. **(A)** Chao 1 index based on the numbers of OTU in the four samples; **(C)** The Principle coordinates analysis (PCoA) score scatter diagram of the four groups. PCoA of the overall diversity based on the Bray Curtis, unweighted UniFrac and weighted UniFrac.; **(B)** The Venn diagrams of the different groups according to bacterial biodiversity; **(D)** Comparison of phylum relative abundance in different groups; **(E,F)** Relative abundances of significantly changed bacterial phyla (Bacteroidetes and Firmicutes); **(G)** The Firmicutes/Bacteroidetes ratio in the different groups. **p < 0.01.

**FIGURE 7:**
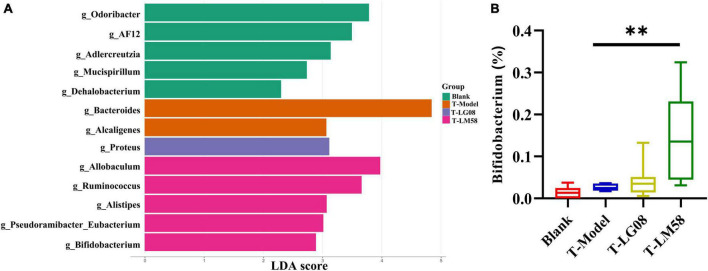
LG08 and LM58 treatment can affect the gut bacterial enrichment. **(A)** LEfSe cladogram of the four groups. **(B)** The relative abundance of *Bifidobacterium* in the four groups. **p < 0.01.

In hyperuricemia rat, the gut microbiota was also evaluated after the therapy of probiotics. Chao1 index in fecal microflora of rat dropped significantly after the treatment with high purine diet and oteracil potassium. And chao 1 increased after LM58 intervention, indicating that hyperuricemia can reduce the abundance of total intestinal flora, and probiotics can promote the recovery of intestinal flora (Figure 6A). Venn analysis showed that T-Model group possessed significantly different structure of the intestinal flora with blank group, T-LG08 and T-LM58 could diminish the effect of oteracil potassium on microbiota changes (Figure 6B). Principle-coordinates analysis (PCoA) results showed that phylogenetic community structures of T-LG08 and T-LM58 groups had no separation from the T-Model group (Figure 6C). Furthermore, relative abundance at the phylum level of each individual sample, *Bacteroidetes* and *Firmicutes* were also the dominant phyla (Figure 6D). However, it seems that the two strains have no influence on the relative abundance of *Bacteroidetes* and induced a reduction in *Firmicutes* (Figures 6E, F). Moreover, the ratio of *Firmicutes* to *Bacteroidetes* was also slightly decreased from 1.1 to 0.95 after the treatment of probiotics (Figure 6G). Surprisingly, from the results of LEfSe cladogram in each group, there was a significant rise in the relative abundance of *Bifidobacterium* in the P-LM58 group compared to the P-Model group (Figures 7A, B). What’s more, *Bifidobacterium* is an extremely important probiotic and has been shown to be associated with the level of serum uric acid levels (Rezazadeh et al., 2021; Gong et al., 2022).

### 3.5. Biosafety evaluation of LG08 and LM08

**FIGURE 8:**
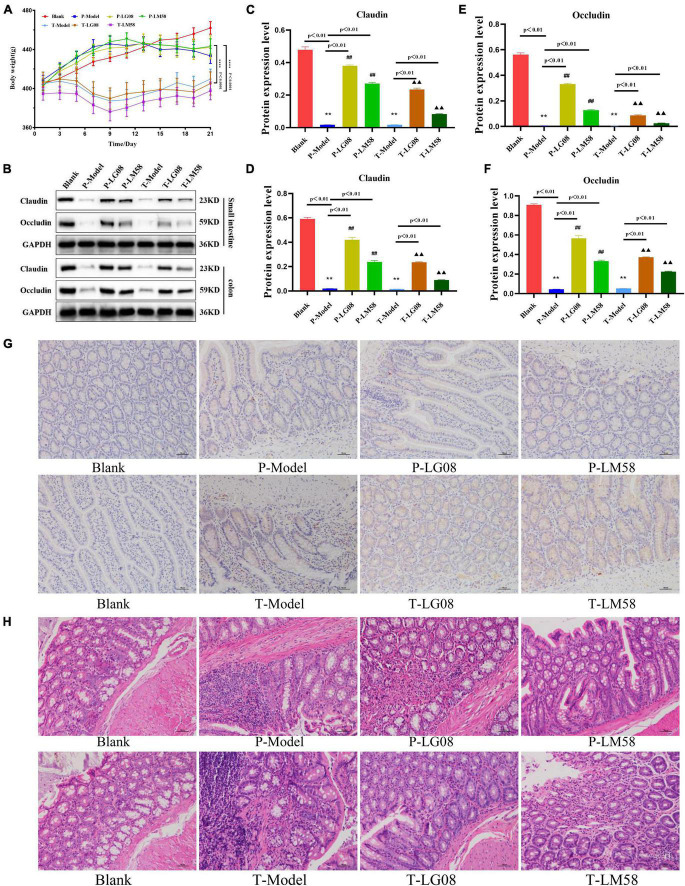
Oral administration of probiotics LG08 and LM58 can alleviate pathological damage caused by hyperuricemia. **(A)** Mice weight changes within 21 days; **(B)** Western blot analysis of proteins expression after different probiotics treatments; **(C,D)** Claudins and occludin proteins expression level in small intestine after LG08 and LM08 prevention; **(E,F)** Claudins and occludin proteins expression level in colon after LG08 and LM08 prevention; **(G)** Immunohistochemistry of endothelial nitric oxide synthase (eNOS) in small intestinal tissues; **(H)** Representative photomicrographs of HE staining of rat colon tissue. **p < 0.01. ^##^*p* < 0.01.

Considering the widespread concern about the biosafety in oral probiotics treatment, *in vivo* biosafety of LG08 and LM08 were further evaluated. During treatment, the body weight was measured every 2 days. As shown in Figure 8A for preventive effect, after oral administration of LG08 and LM08, there was no significant difference between the weight of rat in the probiotic prevention group and the blank group. However, surprisingly, the rats in the treatment group lost significantly more weight than those in the prevention group. These results suggested that the effect of probiotic therapy to regain weight was limited once hyperuricemia has been established, while probiotics could well reduce the damage of the uric acid used as preventive strategy. Furthermore, as a membrane protein within tight junctions, the expression of occludin and claudins proteins play an important role in the maintenance of intestinal mucosal barrier function (Oshima et al., 2003; Günzel and Yu, 2013). Next, the expression of occludin and claudins were further tested by WB assay to evaluate whether oral administration of LG08 and LM08 could repair intestinal mucosa damaged by high uric acid. As indicated in Figure 8B, both the expression of claudin (Figures 8C, D) and occludin (Figures 8E, F) in small intestine and colon were strongly reduced in P-Model and T-model, which represented a serious breach of intestinal integrity. When administrated with probiotics, we found a notable up-regulation of these proteins, especially the LG08 in preventive use. Moreover, the positive acquisition of eNOS expression can also be found after the treatment of probiotics (Figure 8G). Hence, the intestinal mucosal damage caused by oxidative stress of high uric acid can be certainly alleviated upon administrating LG08 and LG58, but the reversible conditions was hard to reach when the hyperuricemia has been established. Finally, the pathological results of the colon showed that the cell space was enlarged and the nuclear staining was deepened in P-Model and T-model groups. In preventive applications, the probiotic intervention appeared to inhibit pathological damage in mucosal cells, which showed no difference in P-LG08 and P-LM58 compared with blank group (Figure 8H).

## 4. Discussion

Hyperuricemia is a metabolic disease caused by abnormal uric acid (UA) metabolism. UA mainly excreted through the kidneys, and about 30% of UA is excreted by the intestine. As the gut microbiota play an increasingly important role in metabolic health of the human host, the gut microbiota has been identified as a new target for hyperuricemia treatment (Wang J. et al., 2022). In our study, we firstly investigated the preventive and therapeutic effects of LG08 and LM58 toward high UA. The results of this investigation showed that the two probiotics played an active part in effective intestinal adhesion and antioxidant activity *in vitro*. Moreover, *in vivo* study demonstrated that the probiotics have the capacity to repair intestinal mucosal and reduce the level of inflammatory and uric acid. Especially, these findings confirmed that both LG08 and LM58 could exert better preventive effect on hyperuricemia to some extent compared with that of the therapeutical use.

Probiotics that can against the clearance of gastric fluid and successfully colonized in the intestine would function well *in vivo*. Thus, the acid and bile salts tolerance of LG08 and LM58 were first measured in our study. The *in vitro* tolerance studies indicated these two strains could display resistance in gut conditions, which is expected to reach the intestine and colon. Then, the adherence ability to intestinal epithelial cells and antagonism against pathogens are also vital factors in effectively adjusting the structure of intestinal flora. The antagonistic effect and adherence ability were evaluated through probiotic surface properties and aggregation ability (Han et al., 2013). Our result indicated that these two strains were less hydrophobic than pathogens, which may contribute to improving the adhesion in intestinal tract. Uric acid is a potent antioxidant and greatly contributes to oxidative stress in the body, and it has been reported that probiotics and their metabolites were responsible for the antioxidant capacity in the host (Uchiyama et al., 2022). Consequently, we found that the supernatant of two strains had a higher antioxidant activity than bacteria cells and the LM58 seems presented slightly stronger antioxidant effect than LG08.

Next, we evaluated if the two probiotics could prevent the development of hyperuricemia in healthy rats. Probiotics alleviate hyperuricemia through various mechanisms. For example, xanthine dehydrogenase (XOD) secreted by *Escherichia coli* is responsible for the oxidative metabolism of purines (Crane, 2013). Besides, some metabolites of probiotics, such as acetate, succinate, and glucose, contribute to providing energy for intestinal epithelial cells to promote uric acid excretion (Nieuwdorp et al., 2014). From the level of biochemical indexes for hyperuricemia in our study, the BUN level was 88% of that in P-Model group for P-LG08 group and 68% of that in P-Model group for P-LM58. Similarly, the CRE and the UA level in serum were in the P-LG08 group were 83 and 94% of those in the P-Model group, while 70 and 75% of those in the P-Model group, respectively. In general, both LG08 and LM58 could effectively prevent the formation of hyperuricemia, especially LM58. In addition, the gut microbiome can affect the process of hyperuricemia by reducing inflammatory reactions and repairing the intestinal barrier. It is noted that the level of inflammatory markers was kept similar to the control after probiotics intervention. What’s more, oral administration of LG08 and LM58 can significantly increase the expression of occludin and claudins proteins, which play an important role in the maintenance of intestinal mucosal barrier function. These results indicated the two strains could effectively suppress the development of hyperuricemia when used as a preventive strategy. However, the effect of these probiotics was not based on increasing the diversity of the gut flora, which is inconsistent with many reports (Wang Z. et al., 2022; Wei et al., 2022). In addition, this also suggested that there is no directly positive correlation between the diversity of intestinal flora and the integrity of intestinal mucosa in high uric acid rat.

Subsequently, we further investigated that whether these probiotics play similar effect after the rat had developed hyperuricemia. As shown in above results, we found although LG08 and LM58 both reverse the symptoms of hyperuricemia to some extent, physiological indicators associated with hyperuricemia were difficult to reduce to the control level. Moreover, once hyperuricemia has developed, the ability of probiotics to reverse and repair antioxidant levels in the body is not apparent. The same phenomenon was also found in influencing intestinal structure. In the safety test, although the state of intestinal cells was improved after treated with probiotics, there was still slight damage compared with the blank group. Thus, a long-term probiotic intervention or combination of drugs may be needed to further repair inflammation and damage in the body.

## 5. Conclusion

To sum up, LM58 showed higher autoaggregation/coaggregation and antioxidant ability *in vitro*, compared to LG08. *In vivo* study, the LM58 decreased the UA levels and regulated the gut microbial structure both in preventive and therapeutic models. On a high-purine diet, the protective effects of LG08 and LM58 against high uric acid were evident, even including reducing inflammation and improving the antioxidant capacity in the body. However, once hyperuricemia is formed, both LG08 and LM58 have a slight effect on the treatment of hyperuricemia, and the oxidative damage caused in the body is also difficult to restore by administration with a single probiotic. In the future, more long-term treatment of probiotic or a combination of drugs may be needed to repair the inflammatory damage caused by high uric acid in the body.

## Data availability statement

The original contributions presented in this study are included in the article/supplementary material, further inquiries can be directed to the corresponding authors.

## Ethics statement

The animal study was reviewed and approved by the Animal Research Ethics Committee of Nanjing University of Chinese Medicine.

## Author contributions

YL, XT, and QH contributed to the conception and design of the study. ZM and FZ performed the statistical analysis. LL wrote the first draft of the manuscript. ZJ, SF, and YL wrote sections of the manuscript. LL, ZM, and FZ contributed equally to the work. All authors contributed to the manuscript revision and read and approved the submitted version.

## Footnotes

1
https://docs.qiime2.org/2019.1/
